# For Exercise, Relaxation, or Spirituality: Exploring Participation Motives and Conformity to Masculine Norms among Male and Female Yoga Participants

**DOI:** 10.3390/ijerph19020770

**Published:** 2022-01-11

**Authors:** Jonathan Y. Cagas, Stuart J. H. Biddle, Ineke Vergeer

**Affiliations:** 1 Department of Sports Science, College of Human Kinetics, University of the Philippines Diliman, Quezon City 1101, Philippines; 2 Physically Active Lifestyles (PALs) Research Group, Centre for Health Research, University of Southern Queensland, Springfield Central 4300, Australia; Stuart.Biddle@usq.edu.au (S.J.H.B.); Ineke.Vergeer@usq.edu.au (I.V.)

**Keywords:** participation motives, yoga, gender differences, holistic movement practice, masculine norms

## Abstract

Yoga is a traditional practice from India with the potential to promote physical activity and health. Participation worldwide remains low, particularly among men. To better understand yoga participation parameters, with a special focus on what influences male participation, this study examined gender differences in participation motives and conformity to masculine norms. It also explored these factors across three participant subgroups who differed in their engagement with the physical and the more psycho-spiritual aspects of yoga. A total of 546 yoga participants (138 males, 399 females, 9 others), 18–73 years old, completed an online survey that included an adapted version of the Exercise Motivation Inventory–2 and three subscales from the Conformity to Masculine Norms Inventory–46. Results showed significant gender differences in participation motives and conformity to masculine norms. Females were more motivated by positive affect, health/fitness, nimbleness, mind–body integration, and coping/stress management, whereas males were more motivated by supplementary activity and competition/social recognition. These differences should be considered in tailoring messages to promote uptake and continued participation. Furthermore, males were more likely than females to conform to emotional control and heterosexual self-presentation masculine norms. Future research may examine how differences in masculine norm adherence influences uptake, particularly among men.

## 1. Introduction

Yoga is a traditional practice from India acknowledged for its potential contribution to public health [1,2]. It involves a holistic approach to health and well-being, promoting physical activity, healthy behaviors, mental health, and psycho-spiritual development [3,4,5,6]. While yoga has become a prominent part of the modern-day physical activity landscape [7,8], studies have shown that yoga participation is still relatively low and appears confined mainly to specific population subgroups [9,10,11,12,13], with white, well-educated females occupying a particularly prominent place. Given yoga’s wide range of potential health benefits [14,15,16,17,18], there is ample space for participation to grow, particularly among non-dominant subpopulations such as men. Strategies for yoga promotion could benefit from knowledge of participation parameters such as people’s perception of the activity and reasons for participation [19,20].

In the physical activity field, yoga can be considered a holistic movement practice [6,7], indicating it involves but also goes beyond physical exercise to include mental as well as spiritual components. Yoga may thus serve various functions, and research has shown that yoga can indeed be perceived in different ways, for example, as a form of physical exercise or as a spiritual activity [21,22,23]. Such perceptions may be associated with the reasons people do or do not do yoga [19,20]. The study of participation motives in the physical activity field in general [24] has shown variations across gender and contexts [20,25,26]. For instance, people who engage in sports are more likely to endorse intrinsic motives such as enjoyment and mastery than those who exercise, whereas people who exercise are more likely to cite health and appearance reasons than sports participants [20,26]. It is also typically reported that men are more likely to exercise or play sports for competitive reasons than women [27,28]. Molanorouzi et al. [20] found that men were driven by competition and improving their skills or personal best when engaging in physical activity. In contrast, women were more motivated by physical appearance and health/fitness.

Similar to physical activity, yoga may be taken up for varying and multiple reasons, including health, fitness, self-development, mind–body integration, and spirituality [4]. Gender differences have been observed in some studies on yoga participation motives [29,30]. A study in Germany [30], for example, found that female participants were more likely to cite spirituality as a reason for participation. Furthermore, findings from a qualitative study involving non-yoga practicing men suggest that men are likely to be less interested in yoga but that they may acknowledge its potential benefits and might be open to taking it up as a supplement to an existing training program [31]. However, not all men share the same needs and interests [32,33], and this may include views on yoga. It is possible that men who consider yoga a psycho-spiritual practice would do yoga for spiritual reasons as much as women. Early research suggests there may be subgroups of participants who differ in their engagement with the psycho-spiritual aspects of yoga [23,34]. It may be useful to take such a possible subdivision into account when considering gender differences in participation motives for yoga.

Gender differences within yoga [35] deserve further study, including examining motives for participation in relation to participants’ perceptions of yoga and their engagement in its psycho-spiritual underpinnings. This study aimed to address this gap, with a particular interest in generating more knowledge about men’s participation motivation, in the hope that this might inform strategies for yoga promotion among this under-represented population subgroup.

Yoga’s potential health benefits [14,15,16,17,18] make it a promising health behavior for both genders. However, as indicated above, uptake among men lags far behind that of women [9,10,11,12] and various factors may contribute to this phenomenon. Men’s health behaviors are considerably influenced by standards of masculinity—existing gender role norms operating in society that expect men to behave in particular ways [36,37,38]. For example, society expects men to be physically and mentally tough, making them less likely to share their emotions, seek help from others, or be concerned about their health [36]. Taking care of one’s health and engaging in alternative health practices like yoga are often associated with the female gender [39,40]. Men who adhere strongly to these masculine norms tend to avoid such practices as a way to maintain their male identity [39]. Strong conformity to traditional masculine standards has been attributed to poor health behaviors among men [39]. Among the many health behaviors, physical activity, particularly sports and strength training programs, tend to be more attractive to men as it reflects masculine values (e.g., physical challenge, being active, and building strength) that are important for men [32,41,42]. Promoting physical activity is, therefore, an important strategy to improve men’s health [32,43]. Studies on health and physical activity promotion among men, however, have predominantly centered on sports to capitalize on these traditional masculine ideals [32]. Yoga is thus less commonly considered as an appropriate activity for men [31]. It has been argued that men have diverse interests and do not conform to masculine norms in similar ways [38,44]. Therefore, some men may not find traditional strength training and sports-centered health programs appealing [45,46,47]. Lack of motivation is a common barrier for physical activity among men, which could result from a lack of variety in the physical activity programs on offer [48]. Thus, exploring other forms of physical activity that could cater to the needs and interests of different male subgroups is important [32,33].

Because of its holistic approach to well-being [7] and preponderance of female participants [12,13], yoga can be perceived as a feminine and female-dominated activity and both those perceptions can act as a barrier for men [31]. However, there are men who practice yoga, and recent data from the United States suggest that their numbers are increasing at least somewhat [10]. In this study, we aimed to get more insight into the motivation of these men by examining participation motives and conformity to masculine norms across both genders. We also wanted to explore the existence and role of different subgroups with respect to these factors, given the research suggesting the existence of participant subgroups differing in their engagement with the psycho-spiritual aspects of yoga [23,34]. Identifying the reasons that best differentiate male and female participants, and those who consider yoga as a physical exercise or a spiritual practice could help develop differentiated messages that effectively target the needs of different participant subgroups. The main research question of this study was whether male and female yoga participants differ in their motives for yoga participation and conformity to certain masculine norms, in general and when considering participant type subgroups.

## 2. Methods

The study consisted of an online survey, including questions on respondents’ socio-demographic background, yoga practice, motives for participation, conformity to masculine norms, perceptions of yoga as a physical, mental, and/or spiritual practice, and immersion in yoga’s psycho-spiritual aspects. This paper particularly focuses on the measures of participation motives, conformity to masculine norms, and a participant type factor representing subgroup differences in engagement with yoga’s psycho-spiritual side, based on the perception and immersion questions.

### 2.1. Participants

Adults, 18 years and older, who were practicing yoga at least once a week for three months prior to the onset of the COVID 19 pandemic were recruited for the study. A minimum sample size of 345 (anticipated effect size = 0.25; power level = 0.80; probability level = 0.05) was determined a-priori using the G*Power program [49]. Study advertisements with a link to the online questionnaire were posted on Facebook, Instagram, Twitter, Yoga Australia’s e-newsletter, and the Australian host University mailing list. Out of the 1119 individuals who accessed the link, 546 (48.8%) completed the survey. Respondents were from the Philippines (*n* = 305; 55.9%), Australia (*n* = 158; 28.9%), and other countries (*n* = 83; 15.2%). Participants’ ages ranged from 18 to 73 years (*M* = 39.99, *SD* =11.85). A majority of respondents were female (73.1%), and Asian (62.5%); 38.5% were married, 47.6% full-time employed, and 44.5% had a bachelor’s degree. Years of yoga practice varied widely from 1 to 50 years (*Mdn* = 5.00 years). The most common styles were Vinyasa Yoga (32.1%) and Ashtanga Yoga (28.3%). The sociodemographic characteristics of the respondents are summarized in Table 1.

### 2.2. Measures

#### 2.2.1. Motives for Yoga Participation

We used the Exercise Motivation Inventory-2 (EMI-2) to assess a broad range of motives and adapted it for yoga participation [50]. The EMI-2 includes 51 items measuring 14 participation motives. We added 28 new items to capture six additional motives (Spirituality, Mind–Body Integration, Centering/Mindfulness, Personal Growth/Transformation, supplementary activity, and general coping) that may be salient to yoga [4]. Details of the process of developing these additional items and the preliminary factor analysis are reported elsewhere [51]. The exploratory factor analysis of the adapted EMI-2 resulted in 12 factors: Spirituality (e.g., “for spiritual growth”), Mind–Body Integration (e.g., “to have a better mind-body connection”), Coping/Stress Management (e.g., “to better cope with some challenging life events”), Health/Fitness (e.g., “to feel more healthy”), Nimbleness (e.g., “to maintain flexibility”), Challenge (e.g., “to give me goals to work towards”), Positive Affect (e.g., “because it makes me feel good”), Weight Management/Appearance (e.g., “to lose weight”), Ill-Health Avoidance (e.g., “to prevent health problems”), Affiliation (e.g., “to spend time with friends”), Competition/Social Recognition (e.g., “to show my worth to others”), and Supplementary Activity (e.g., “because it will help me in my sport or other physical activity”). Internal reliability coefficients were high, ranging from 0.85 to 0.97. Participants indicated whether or not each statement was true for them using a 6-point Likert-type scale ranging from 0 (Not at all true for me) to 5 (Very true for me).

#### 2.2.2. Conformity to Masculine Norms

The Conformity to Masculine Norms Inventory-46 [52,53] assesses the extent to which an individual adheres to nine traditional masculine norms. Owen [54] suggested selecting only the masculine norms that may be salient to the topic. In this study, we assessed only three norms: Emotional Control (e.g., “I tend to keep my feelings to myself”), Heterosexual Self-Presentation (e.g., “I would feel uncomfortable if someone thought I was gay”), and Self-Reliance (e.g., “I never ask for help”). Internal reliability coefficients of these three subscales ranged from 0.85 to 0.87. Because of the feminine and female-dominated nature of yoga [31], we believed these norms would be the most relevant. Participants indicated the extent to which they agreed or disagreed with each statement using a 4-point scale ranging from 0 (strongly disagree) to 3 (strongly agree). Means for each subscale were computed, with higher scores reflecting greater conformity.

#### 2.2.3. Yoga Participant Subgroups

Participants were categorized into *Exercisers*, *Yogis*, and *Postural Yogis*. This categorization was based on a two-step cluster analysis on three clustering variables: perceptions of yoga as a physical practice, perceptions of yoga as a psycho-spiritual practice, and yoga immersion (i.e., level of engagement in the psycho-spiritual aspects of yoga) [55]. Details of the cluster analysis are reported elsewhere [51]. *Exercisers* were yoga participants who perceived yoga mainly as a physical practice and were the least involved in yoga’s psycho-spiritual dimensions. *Yogis* were participants who regarded yoga more as a psycho-spiritual discipline and less as a physical practice; they were highly immersed in yoga’s psycho-spiritual teachings. *Postural Yogis* were those who viewed yoga both as a physical practice and a psycho-spiritual discipline and were also highly involved in its psycho-spiritual aspects.

### 2.3. Data Analyses

Preliminary data screening was performed prior to the main analyses [56]. To examine differences in participation motives and conformity to masculine norms across gender and participant type, two multivariate analyses of covariance (MANCOVA) were conducted. In the first analysis, participation motives were entered as dependent variables with gender and participant type as independent variables, and age, years of practice, and teacher status as covariates. This analysis was repeated without the covariates. When appropriate, a discriminant function analysis (DFA) was conducted as a follow-up on the main effects. Multiple group comparisons with Bonferroni corrections were used for the subgroup analyses. The same procedure was followed in the second analysis but with the three conformity to masculine norms subscales as dependent variables. All statistical analyses were executed using IBM SPSS Statistics for Macintosh, version 27.0 (IBM Corp., Armonk, NY, USA) [57].

## 3. Results

Seven multivariate outliers (Mahalanobis’ distance values > 32.91, *p* = 0.001) were identified and removed from the analyses. Overall, the top motives for yoga participation were Positive Affect (*M* = 4.19, *SD* = 0.96), Health/Fitness (*M* = 4.14, *SD* = 0.95) and Nimbleness (*M* = 4.14, *SD* = 1.06). Competition/Social Recognition (*M* = 0.98, *SD* = 1.07) was the least endorsed motive.

### 3.1. Gender Differences in Participation Motives

Results from the MANCOVA (*n* = 530) indicated significant main effects for gender, Pillai’s Trace = 0.137, *F* (12, 510) = 6.733, *p* < 0.001, *ηp*^2^ = 0.137; and gender by participant type interaction, Pillai’s Trace = 0.093, *F* (24, 1022) = 2.081, *p* < 0.01, *ηp*^2^ = 0.047. The three covariates were also significant: Age, Pillai’s Trace = 0.118, *F* (12, 510) = 5.66, *p* < 0.001, *ηp*^2^ = 0.118; Years of Practice, Pillai’s Trace = 0.07, *F* (12, 510) = 3.178, *p* < 0.001, *ηp* ^2^ = 0.07; and Teacher Status, Pillai’s Trace = 0.046, *F* (12, 510) = 2.055, *p* < 0.05, *ηp*^2^ = 0.046 (Supplementary Tables S2–S8). Age correlated positively with Nimbleness and negatively with Positive Affect, Mind–Body Integration, Coping/Stress Management, Spirituality, Challenge, Weight Management/Appearance, Supplementary Activity, and Competition/Social Recognition. When gender was considered, the relationships between Age and Positive Affect, Nimbleness, Challenge, and Competition/Social Recognition were significant only for women, and Spirituality and Weight Management/Appearance were significant only for men. Years of Practice correlated positively with Spirituality, and negatively with Health/Fitness, Challenge, Weight Management/Appearance, Supplementary Activity, and Competition/Social Recognition. When gender was considered, the relationships between Years of Practice and motives such as Spirituality, Weight Management/Appearance, and Competition/Social Recognition were significant only for women, whereas Supplementary Activity was significant only for men. Teachers reported higher Positive Affect, Mind–Body Integration, Coping/Stress Management, Ill-Health Avoidance, and Spirituality than Non-Teachers, whereas Non-Teachers reported higher Nimbleness than Teachers. These differences were also observed when the data was split by gender, except for three motives. Male teachers rated Positive Affect, Ill-health Avoidance, and Affiliation higher than male non-teachers. No significant differences were observed in these motives between female teachers and female non-teachers. The same significant result was obtained when the analysis was repeated without the covariates (i.e., MANOVA), Pillai’s Trace = 0.0137, *F* (12, 513) = 6.782, *p* < 0.001, *ηp* ^2^ = 0.137.

Pairwise comparisons revealed significant gender differences in Positive Affect (*p* < 0.001), Health/Fitness (*p* < 0.001), Nimbleness (*p* < 0.05), Mind–Body Integration (*p* < 0.001), Coping/Stress Management (*p* < 0.001), Supplementary Activity (*p* < 0.01), and Competition/Social Recognition (*p* < 0.05). Inspecting the means (Table 2) indicated that female participants rated Positive Affect, Health/Fitness, Nimbleness, Mind–Body Integration, and Coping/Stress Management higher than male participants, whereas male participants rated Supplementary Activity and Competition/Social Recognition higher than their female counterparts.

These differences were further supported by a discriminant function analysis (DFA) with prior probabilities computed from the actual group sizes, which revealed a significant canonical function, Wilks’ λ = 0.87, χ2 (12) = 74.66, *p* < 0.001. This result indicates that male and female yoga participants could be discriminated effectively by the motives, with 75.6% of cases correctly classified into gender. Based on a minimum discriminant function loading of 0.30 [56], Coping/Stress Management (−0.635), Positive Affect (−0.580), Mind–Body Integration (−0.500), and Health/Fitness (−0.487) contributed most to gender differences [58], with Supplementary Activity (0.272), Nimbleness (−0.251), and Competition/Social Recognition (0.241) providing a small contribution.

### 3.2. Gender, Participant Subgroups, and Participation Motives

As indicated in Table 1, about a quarter (24.2%) of the respondents were classified as Exercisers. The rest was evenly divided among Yogis (37.4%) and Postural Yogis (38.5%). Within gender, the percentage Yogis was similar in both gender groups at about 37%. Although the percentage Exercisers was higher among males (29.0%) than among females (22.6%) and the percentage Postural Yogis was lower among males (34.1%) than among females (40.1%), the test of proportions showed no significant association between gender and participant subgroups, χ^2^ (2) = 2.01, *p* = 0.37.

#### 3.2.1. Gender Differences in Participation Motives within Participant Subgroups

**Figure 1:**
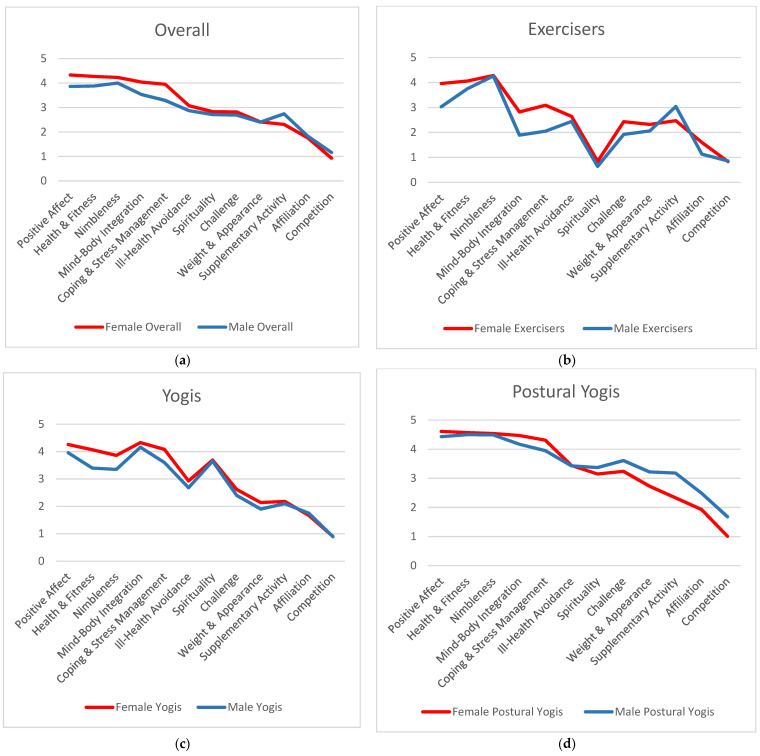
Mean scores on participation motives per gender, for (**a**) all participants, (**b**) Exercisers subgroup, (**c**) Yogis subgroup, and (**d**) Postural Yogis subgroup.

Multiple group comparisons with Bonferroni corrections revealed significant gender differences in participation motives within participant groups (Figure 1 and Table 2). Within the Exercisers subgroup, gender differences were found in Positive Affect, Mind–Body Integration, Coping/Stress Management, and Challenge, with male Exercisers rating these motives lower than female Exercisers. Within the Yogis subgroup, gender differences were observed in Positive Affect, Health/Fitness, Nimbleness, and Coping/Stress Management, with male Yogis rating these motives lower than female Yogis. Within Postural Yogis, gender differences were identified in Mind–Body Integration, Coping/Stress Management, Weight Management/Appearance, Supplementary Activity, Affiliation, and Competition/Social Recognition. Male Postural Yogis rated Mind–Body Integration and Coping/Stress Management lower, and Weight Management/Appearance, Supplementary Activity, Affiliation, and Competition/Social Recognition higher than female Postural Yogis.

#### 3.2.2. Participation Motives within Participant Subgroups per Gender

**Figure 2:**
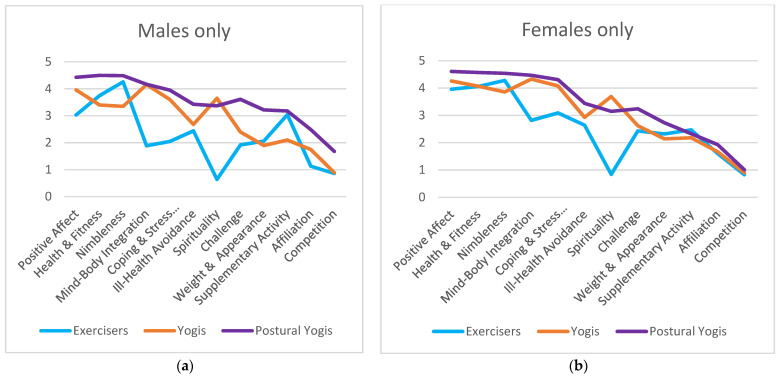
Mean scores on participation motives per participant subgroup, for (**a**) males only and (**b**) females only.

Significant differences were also found within gender subgroups. Among males (Figure 2a and Supplementary Table S10), the three participant subgroups differed significantly in all motives. Postural Yogis and Exercisers differed significantly in most motives but two. Postural Yogis scored higher than Exercisers on all motives except for Nimbleness and Supplementary Activity, where both subgroups scored similarly. Postural Yogis and Yogis also differed significantly on almost all motives, with Postural Yogis scoring higher on most motives except for Positive Affect, Mind–Body Integration, and Coping/Stress Management, where the scores were similar, and Spirituality, where the Yogis scored higher. Yogis and Exercisers differed on half of the motives, with Yogis scoring higher than Exercisers on Positive Affect, Mind–Body Integration, Coping/Stress Management, and Spirituality, and lower on Nimbleness and Supplementary Activity.

Among females (Figure 2b and Supplementary Table S11), the three participant subgroups differed significantly in most motives except in Supplementary Activity, Affiliation, and Competition/Social Recognition, where the scores were similar. Postural Yogis scored higher than Exercisers in all other motives except Nimbleness and Weight Management/Appearance, where the scores were not statistically different. Postural Yogis scored higher than Yogis in all other motives except in Mind–Body Integration and Coping/Stress Management, where the scores were similar, and in Spirituality, where yogis scored higher than Postural Yogis. Yogis scored higher than Exercisers in five motives (Positive Affect, Mind–Body Integration, Coping/Stress Management, and Spirituality) and lower in one motive (Nimbleness). Both Yogis and Exercisers scored similarly in Health/Fitness, Ill-Health Avoidance, and Challenge motives.

### 3.3. Gender, Participant Subgroups, and Conformity to Masculine Norms

#### 3.3.1. Gender Differences in Conformity to Masculine Norms

Results from the second MANCOVA indicated significant main effects for gender, Pillai’s Trace = 0.021, *F* (3, 519) = 3.794, *p* < 0.01, *ηp*^2^ = 0.021. The gender by participant type interaction, however, was not significant. The three covariates (age, teacher status, and years of practice) were also not significant. The same significant result was obtained when the analysis was repeated without the covariates, Pillai’s Trace = 0.024, *F* (3, 522) = 4.227, *p* < 0.01, *ηp*^2^ = 0.024.

Pairwise comparisons showed male yoga participants reported higher Emotional Control, *p* = 0.008, and Heterosexual Self-Presentation, *p* = 0.027 compared to female yoga participants (Table 3). However, no significant gender difference was found in Self-Reliance, *p* = 0.604.

#### 3.3.2. Gender Differences in Conformity to Masculine Norms within Participant Subgroups

When yoga participant subgroups were examined separately, male Yogis reported higher Emotional Control than female Yogis, *t* (196) = 2.532, *p* = 0.012, and male Postural Yogis reported higher Heterosexual Self-Presentation than female Postural Yogis, *t* (203) = 2.381, *p* = 0.018. When examining gender separately, no significant differences were found across the participants subgroups for either gender.

## 4. Discussion

The present study examined whether participation motives and conformity to certain masculine norms vary across gender and type of yoga participants. Results indicated that, overall, participation motivation in yoga and conformity to masculine norms differed significantly between the genders. Males were more motivated than females to do yoga as a supplement for their other sports or physical activities and to display competitiveness. In contrast, females were more motivated than males by mind-body integration, health/fitness, positive affect, nimbleness, and coping/stress management. There were, however, variations in these differences when comparing participant subgroups. Coping/stress management most consistently distinguished the genders, followed by positive affect, mind-body integration, and supplementary activity, which distinguished the genders in two of the three subgroups. With respect to masculine norms, male yoga participants were more likely to demonstrate emotional control and present themselves as heterosexual compared with female yoga participants. Overall, the results suggest that it may be important to differentiate yoga messages for increased participation. Particularly when encouraging men to participate, different aspects may need to be emphasized. The results also highlight the potential role of masculinity in men’s participation experience in yoga.

### 4.1. Differences in Participation Motives

Research has consistently shown that differentiation in motives underlie men and women’s participation in physical activity, including yoga [20,30,59]. This differentiation in motives was also reflected in our findings. More specifically, our results indicate that compared to women, men are more likely to do yoga as a supplementary activity and for competitive and social recognition reasons, and less likely to cite motives related to mind-body integration, health/fitness, positive affect, nimbleness, and coping/stress management. Doing yoga to supplement their primary sport or physical activity, including injury recovery, was a salient motive for men, which is in line with previous studies [4,31]. It is, however, noteworthy that there were no gender differences in ill-health avoidance and weight management/appearance motives. Previous studies have typically found these types of motives to be higher among females [20,60]. This may imply that the nature of yoga attracts not only women but also men who seek such physical benefits from yoga. For male Postural Yogis, weight and appearance motives were even higher than for female Postural Yogis, suggesting that this was a particular subgroup of men valuing physical health and appearance effects.

For both genders, but particularly so for males, Postural Yogis had the highest scores on all motives (with the exception of spirituality, where Yogis scored higher, but this was still a fairly important motive for Postural Yogis), suggesting that this subgroup was most strongly and most widely motivated. The group of Postural Yogis was proportionally smaller among males than among females, suggesting that this combination of wide interests may be less common among male yoga participants. To what extent this reflects the presence of this subgroup in the wider male population remains to be investigated, along with possible other characteristics of this subgroup of men. However, it suggests there is a subgroup of men who feel attracted by both physical and non-physical components of yoga.

Although men’s motivation to practice physical activity seems inconsistent with the non-competitive and non-performance principles of yoga, yoga still falls within the domain of physical activity, providing men with opportunities to demonstrate traditional masculine ideals, such as comparing their abilities with others and displaying their physical strength [41,61]. Highlighting the potential of yoga to supplement sports and exercise may therefore appeal better to men. However, not all men are necessarily attracted to these conventional activities [45,46]. For example, two studies involving male yoga participants reported that one reason why men practiced yoga was that they found yoga more meaningful compared to sport and other conventional physical activities [62,63]. It is possible that the men in these studies particularly represented Yogis and Postural Yogis, who value not only health benefits but also opportunities for self-inquiry and personal transformation. Future research, however, needs to investigate this further as we did not ask participants whether yoga was their primary form of physical activity. Future research may also investigate whether different types of male yoga practitioners come with different needs and/or derive different benefits from yoga. Competition was the lowest motive for all female subgroups and one of the lowest motives for all male subgroups (the lowest for Yogis and Postural Yogis, and the second lowest for Exercisers), reflecting the non-competitive nature of yoga. This confirms that yoga may be a good alternative form of physical activity for men who are not attracted to traditional competitive forms of physical activity. At the same time, yoga could appeal to men who do have competitive interests in sports or exercise as a form of supplementary activity.

In contrast to previous studies suggesting that men are less likely than women to cite spirituality as a motive for yoga participation [30], our results showed no significant gender difference in this motive. Differences in the importance of spirituality were found between yoga participant subgroups, rather than between the genders. Spirituality was an important motive for both male and female Yogis and Postural Yogis, and the least important motive for Exercisers regardless of gender. Yoga, by origin, is a psycho-spiritual discipline and thus includes spiritual practices such as chanting and rituals [64]. While these practices could act as barriers to those who hold strong religious beliefs, they could also facilitate participation in those who are already interested in spirituality and other traditions [4,65].

### 4.2. Masculinity, Femininity, and Masculine Norms

The holistic spirituality and well-being associated with yoga generally fit with traditional feminine ideals [40,66], which is a reason why yoga is often stereotyped as a feminine activity. This stereotyping could act as a barrier to participation to some men, especially if they hold strong traditional masculine beliefs [67].

Society generally expects men and women, to behave in particular ways and engage in gender-appropriate activities [36]. As a result, men and women avoid engaging in activities that contradict these gender expectations, as these could lead to gender role conflicts and adverse psychological outcomes (e.g., self-devaluation) [68]. The socio-cultural contexts influencing men’s decision to pursue or avoid certain types of physical activities, therefore, cannot be ignored. However, men do not belong to a single homogenous group and may express their masculinities in various ways [69,70,71]. Studies on alternative health practices suggest men learn to navigate this potential conflict by using instrumental reasons (e.g., to recover from injuries) and downplay the ‘feminine’ aspects of such practices (e.g., discussing health and emotions) to justify their involvement in these socially perceived feminine activities [72,73].

As anticipated and consistent with the broader literature on masculine norms [74], the results indicated that male yoga participants were more likely to adhere to masculine norms compared to female yoga participants. More specifically, male yoga participants were more concerned about expressing emotions and about being thought of as gay than female yoga participants, although the genders did not differ on self-reliance. The feminine and female-dominated nature of yoga may thus be challenging even to males who have found their way into yoga, but it is possible that men who practice yoga are able to alter the feminine-coded practice of yoga by using logic and rationality to explain their participation, similar to other men who engage in other feminine-stereotyped activities such as vegetarianism/veganism [75], complementary and alternative medicine [73], or health [76].

It is worth noting that when gender differences were examined within participant subgroups, only male Yogis reported higher conformity to emotional control, and only male Postural Yogis reported higher conformity to heterosexual self-presentation than their female counterparts. The experience and control of emotions is an interesting phenomenon in the context of yoga. Studies have shown that that conformity to the masculine norm of emotional control may act as a protective buffer helping men to avoid anger and stress [77], and other maladaptive behaviors, such as binge drinking [78]. The practice of yoga involves stress management techniques, such as relaxation, breathing, and meditation, promoting emotional self-regulation [79]. Therefore, it is possible that with long-term practice, the type of control that men use may shift from suppressing to recognizing and internally regulating their emotions. This would serve the masculine norm of not expressing emotions in a healthier way, and this may be what underlies the higher scores of the male Yogis. In contrast, female Yogis may learn to express a broader range of emotions and cope better when talking to others. Males and females have been shown to cope with stress differently, with men more likely to use emotional inhibition than women while women are more likely to use emotional coping than men [80]. An interesting direction for future research would be to explore what happens to emotion regulation with prolonged yoga practice, and to what extent this affects typical gender differences in emotional control.

While conformity to the masculine norms of emotional control and heterosexual self-presentation was stronger for male than for female participants, there were no differences in adherence to masculine norms between the participant subgroups, neither among females nor among males. The fact that conformity to masculine norms did not differ among the male subgroups suggests that, generally, the pressure to conform to masculine norms is present among all yoga-practicing men. An interesting question is to what extent the conformity to masculine norms of male yoga participants differs from conformity to masculine norms by other groups of men. A brief perusal of the literature shows that the mean scores of the three conformity to masculine norms dimensions in the present study were comparable to those reported in a study involving American men [53], but were higher than those reported in studies involving predominantly Caucasian men [81,82,83] and Asian Americans [82]. Future studies will need to directly compare conformity to masculine norms between yoga-participating and non-yoga participating men, and may also investigate how yoga-participating men construe their masculinity within a (currently) female-dominated context, and how they overcame any barriers associated with the feminine and female-dominated nature of yoga.

Another noticeable gender difference in this study was the fact that a third of the male respondents were yoga teachers compared to only 6% of the female respondents. Whether this is an artefact of a stronger tendency of male yoga teachers (compared to male participants) to respond to the survey or an indication that men who do find their way to yoga are more likely than women to become teachers is an intriguing question that deserves further study.

### 4.3. Implications for Yoga Promotion among Men

The results suggest that men might be more receptive to yoga if it is promoted as a supplementary activity or rehabilitation modality. Studies in the United States have shown that yoga is one of the most commonly used complementary approaches to improve athletic performance [84], and it is also one of the most commonly recommended complementary health approaches by general practitioners [85]. Male participants have remained a minority in yoga in most Western societies [12,35,86,87]. Therefore, encouraging more general practitioners to promote yoga to their male patients as a supplementary activity may be a useful strategy to help men be more receptive to yoga. There is undoubtedly room for increased participation among men as yoga also has the potential to promote healthier lifestyle behaviors [88,89]. Highlighting how yoga could help men improve in their primary physical activity or enhance their athletic performance might encourage increased uptake. For example, yoga classes that focus on mobility, core stability, and flexibility may be more enticing for men. Although it is generally acknowledged that increasing awareness does not necessarily result in a change in behavior [90], one study reported that non-yoga practicing men found brief information sessions helpful in making them understand the principles behind yoga and what other benefits it could offer [31]. Providing short information sessions or optional men-only introductory classes may be useful in increasing yoga uptake among men.

In both male and female participants, spirituality and mind-body integration were motives that distinguished Yogis from Postural Yogis and Exercisers, and Postural Yogis from Exercisers. As demonstrated in a previous qualitative study [23,34], some yoga participants do yoga for spiritual reasons and have deeper engagement in its psycho-spiritual aspects. The present results indicate that, similar to women, some men are motivated to do yoga primarily for health and fitness reasons while others are driven by spirituality, self-exploration, and personal growth. Promoting yoga to men may therefore depend on what men seek. On one hand, the potential of yoga as a supplementary training program for sports can be emphasized when promoting yoga to men who are seeking alternative forms of physical exercise or physical therapy. Highlighting the spiritual and mind-body sides of yoga, on the other hand, may also be helpful to capture men who are more interested in benefits beyond physical exercise and performance. Acknowledging differentiation among men is therefore essential to promote increased yoga uptake.

### 4.4. Strengths and Limitations

While this study may be the first to demonstrate gender differences in participation motives and conformity to masculine norms in various yoga participant subgroups, some limitations need to be noted. First, the cross-sectional design does not allow for an analysis of how participation motives may change over time or how conformity to masculine norms influenced participation adherence. Future investigations should employ longitudinal designs to investigate mechanisms that drive changes in motivation and adherence to traditional masculinity norms and how these changes influence yoga participation uptake and maintenance. Second, the use of self-report data increased the likelihood of social desirability bias. Future studies could include measures to minimize this or use mixed methods designs to provide more diverse and robust data. Third, self-selection bias is possible due to the online survey design, recruitment, and data collection method. We exerted effort to recruit across a wide range of participants (e.g., demographics and practice styles). Nevertheless, we acknowledge that our study participants were highly educated, mostly Vinyasa and Ashtanga yoga practitioners, and more than half coming from the Philippines. The physical activity landscape in the Philippines is strongly influenced by the United States [91,92]. While we recognize the potential influence of socio-cultural factors, the way people understand and practice yoga in the Philippines is likely to be comparable to those from other Western societies. Future investigations would benefit by replicating the present study in different populations to extend its generalizability. Fourth, we acknowledge the unequal distribution of male and female participants. While we tried to recruit as many male yoga participants as possible, the challenge was that they were simply outnumbered by females, as reflected in previous studies [12,13]. Finally, this study used only three subscales of the Conformity to Masculine Norms Inventory. While researchers support the use of selected masculine norms that are salient in a particular investigation [53,93], future studies should consider using the full spectrum of the scale to allow for a more comprehensive understanding of the role of masculinity in yoga participation, particularly among men.

## 5. Conclusions

This study examined the extent to which participation motives and conformity to certain masculine norms vary across gender and subgroups of yoga participant types. The findings point to the importance of differentiated messaging in yoga to capture a wider male audience. Although the top motives for yoga participation were consistent with previous studies, there were significant differences across gender, with male participants more likely to endorse taking up yoga as a supplementary activity and for competition and social recognition but less likely to cite coping and stress management, positive affect, mind-body integration, and health and fitness. When participant subgroups were considered, holistic motives were salient reasons even for male participants. The results of this study add to our understanding that not all male yoga participants are merely interested in yoga for its health and fitness benefits. Some male yoga participants do consider yoga more than just physical exercise and are interested in its psycho-spiritual teachings. These are important considerations when promoting yoga among men.

## Figures and Tables

**Table 1:** Characteristics of the participants.

Variables	Total	Male	Female	Others *
**Sample size**	546	138 (25.3%)	399 (73.1%)	9 (1.6%)
**Age**				
Mean (SD)	39.99 (11.85)	41.94 (12.09)	39.43 (11.76)	35.00 (7.75)
Range	18 to 73 years	21 to 69 years	18 to 73 years	26 to 47 years
**Country of Residence**				
Australia	158 (28.9%)	42 (30.4%)	114 (28.6%)	2 (22.2%)
Philippines	305 (55.9%)	57 (41.3%)	242 (60.7%)	6 (66.7%)
Others	83 (15.2%)	39 (28.3%)	43 (10.8%)	1 (11.1%)
**Ethnicity**				
White	172 (31.5%)	59 (42.8%)	113 (28.3%)	0 (0.0%)
Asian	341 (62.5%)	66 (47.8%)	268 (67.2%)	7 (77.8%)
Mixed-race	13 (2.4%)	5 (3.6%)	7 (1.8%)	1 (11.1%)
Others	20 (3.7%)	8 (5.8%)	11 (2.8%)	1 (11.1%)
**Employment**				
Employed full-time	260 (47.6%)	70 (50.7%)	186 (46.6%)	4 (44.4%)
Employed part-time	46 (8.4%)	9 (6.5%)	36 (9.0%)	1 (11.1%)
Self-employed	139 (25.5%)	38 (27.5%)	99 (24.8%)	2 (22.2%)
Studying full-time	35 (6.4%)	6 (4.3%)	27 (6.8%)	2 (22.2%)
Retired	20 (3.7%)	10 (7.2%)	10 (2.5%)	0 (0.0%)
Stay at home parent/carer	16 (2.9%)	0 (0.0%)	16 (4.0%)	0 (0.0%)
Unemployed	15 (2.7%)	2 (1.4%)	13 (3.3%)	0 (0.0%)
Other	15 (2.7%)	3 (2.2%)	12 (3.0)	0 (0.0%)
**Partnership Status**				
Single	180 (33.0%)	46 (33.3%)	130 (32.6%)	4 (44.4%)
Partnered or in a relationship	125 (22.9%)	40 (29.0%)	83 (20.8%)	2 (22.2%)
Married/Civil partnership/De facto	210 (38.5%)	46 (33.3%)	161 (40.4%)	3 (33.3%)
Divorced/separated	28 (5.1%)	5 (3.6%)	23 (5.8%)	0 (0.0%)
Other (e.g., widower)	3 (0.5%)	1 (0.7%)	2 (0.5%)	0 (0.0%)
**Highest Educational Attainment**				
Less than Year 12 or equivalent	6 (1.1%)	1 (0.7%)	5 (1.3%)	0 (0.0%)
Year 12 or High School diploma	29 (5.3%)	6 (4.33%)	22 (5.5%)	1 (11.1%)
Vocational or Associate Diploma	40 (7.3%)	16 (11.6%)	22 (5.5%)	2 (22.2%)
Bachelor	243 (44.5%)	51 (37.0%)	190 (47.6%)	2 (22.2%)
Postgraduate diploma/certificate	60 (11.0%)	11 (8.0%)	48 (12.0%)	1 (11.1%)
Master’s degree	122 (22.3%)	39 (28.3%)	80 (20.1%)	3 (33.3%)
Doctorate	46 (8.4%)	14 (10.1%)	32 (8.0%)	0 (0.0%)
**Socio-Economic Ladder**				
Mean (SD)	6.21 (1.72)	6.21 (1.76)	6.21 (1.71)	6.33 (1.73)
Range	1–10	1–10	1–10	4–9
**Yoga Teacher Status**				
Non-teachers	397 (72.7%)	93 (67.4%)	297 (74.4%)	7 (77.8%)
Teachers	149 (27.3%)	45 (32.6%)	25 (6.%)	2 (22.2%)
**Subgroups of Yoga Participant Type**				
Exerciser	132 (24.2%)	40 (29.0%)	90 (22.6%)	2 (22.2%)
Yogi	204 (37.4%)	51 (37.0%)	149 (37.3%)	4 (44.4%)
Postural Yogi	210 (38.5%)	47 (34.1%)	160 (40.1%)	3 (33.3%)

* Not included in the group comparison due to a very small sample size.

**Table 2:** Descriptive statistics of participation motives by gender and participant types.

	**Overall**		**Exercisers**		**Yogis**		**Postural Yogis**	
*n* (%)	530 (100%)		127 (24%)		198 (37.3%)		205 (38.7%)	
	**Female**	**Male**	**Female**	**Male**	**Female**	**Male**	**Female**	**Male**
*n* (%)	394 (74.3%)	136 (25.7%)	89 16.8%)	38 (7.2%)	147 (27.7%)	51 (9.6%)	158 (29.8%)	47 (8.9%)
**Motives**	**M (sd)**	**M (sd)**	**M (sd)**	**M (sd)**	**M (sd)**	**M (sd)**	**M (sd)**	**M (sd)**
Positive Affect	4.33 *** (0.77)	3.86 (1.18)	3.96 *** (0.89)	3.03 (1.38)	4.26 * (0.76)	3.96 (1.01)	4.61 (0.58)	4.43 (0.7)
Health/Fitness	4.27 *** (0.84)	3.88 (1.03)	4.06 (0.93)	3.75 (0.81)	4.07 *** (0.91)	3.4 (1.18)	4.57 (0.61)	4.5 (0.62)
Nimbleness	4.23 * (0.99)	4 (1.11)	4.28 (0.86)	4.26 (0.85)	3.86 ** (0.19)	3.35 (1.29)	4.54 (0.7)	4.49 (0.67)
Mind–Body Integration	4.04 *** (1.08)	3.53 (1.45)	2.82 *** (1.23)	1.89 (1.37)	4.33 (1.74)	4.16 (1.86)	4.47 * (0.68)	4.17 (0.87)
Coping/Stress Management	3.95 *** (1.07)	3.29 (1.46)	3.09 *** (1.14)	2.05 (1.52)	4.08 ** (1.94)	3.60 (1.09)	4.31 * (0.86)	3.95 (1.11)
Ill-Health Avoidance	3.07 (1.31)	2.87 (1.38)	2.64 (1.25)	2.44 (1.31)	2.93 (1.31)	2.68 (1.29)	3.44 (1.26)	3.43 (1.36)
Spirituality	2.83 (1.66)	2.71 (1.84)	0.84 (0.85)	0.64 (1.03)	3.69 (1.24)	3.65 (1.22)	3.15 (1.43)	3.37 (1.59)
Challenge	2.82 (1.27)	2.69 (1.49)	2.43 * (1.16)	1.92 (1.36)	2.62 (1.28)	2.4 (1.45)	3.24 (1.22)	3.61 (1.11)
Weight Management/Appearance	2.41 (1.46)	2.4 (1.5)	2.32 (1.45)	2.06 (1.51)	2.14 (1.43)	1.9 (1.29)	2.73 (1.45)	3.22 * (1.39)
Supplementary Activity	2.31 (1.78)	2.74 * (1.72)	2.47 (1.71)	3.04 * (1.51)	2.18 (1.74)	2.1 (1.6)	2.33 (1.85)	3.18 ** (1.84)
Affiliation	1.76 (1.40)	1.83 (1.56)	1.60 (1.39)	1.13 (1.35)	1.67 (1.39)	1.75 (1.48)	1.93 (1.39)	2.49 * (1.57)
Competition/Social Recognition	0.93 (1.06)	1.16 * (1.27)	0.83 (0.91)	0.86 (1.06)	0.90 (1.01)	0.89 (1.06)	1.01 (0.98)	1.68 *** (1.48)

*** Significant difference between the genders: *** *p* < 0.001. ** *p* < 0.01; * *p* < 0.05.

**Table 3:** Descriptive statistics of Conformity to Masculine Norms subscales by gender and participant types.

	Overall		Exercisers		Yogis		Postural Yogis	
	Male	Female	Male	Female	Male	Female	Male	Female
Masculine Norm	M (sd)	M (sd)	M (sd)	M (sd)	M (sd)	M (sd)	M (sd)	M (sd)
Emotional Control	2.36 ** (0.56)	2.19 (0.58)	2.39 (0.70)	2.32 (0.63)	2.40 * (0.49)	2.18 (0.54)	2.30 (0.50)	2.13 (0.58)
Self-Reliance	2.17 (0.53)	2.13 (0.60)	2.14 (0.46)	2.19 (0.59)	2.22 (0.55)	2.15 (0.58)	2.14 (0.56)	2.07 (0.61)
Heterosexual Self-Presentation	1.8 * (0.61)	1.66 (0.57)	1.78 (0.68)	1.71 (0.68)	1.79 (0.53)	1.69 (0.54)	1.83 * (0.65)	1.61 (0.52)

*** Significant difference between the genders: *** *p* < 0.001. ** *p* < 0.01; * *p* < 0.05.

## Data Availability

The data that support the findings of this study are available from the corresponding author, J.Y.C., upon reasonable request.

## Supplementary Materials

The following are available online at https://www.mdpi.com/article/10.3390/ijerph19020770/s1, Table S1: Structure coefficients for gender canonical function, Table S2: Correlations between age and participation motives (Overall), Table S3: Correlations between age and participation motives (Males), Table S4: Correlations between age and participation motives (Females), Table S5: Correlations between years of practice and participation motives (Overall), Table S6: Correlations between years of practice and participation motives (Males), Table S7: Correlations between years of practice and participation motives (Females), Table S8: Descriptive statistics of Conformity to Masculine Norms subscales by gender and participant subgroups, Table S9: Differences in participation motives across participant subgroups (Overall), Table S10: Differences in participation motives across participant subgroups (Males), and Table S11: Differences in participation motives across participant subgroups (Females).
