# Supplementary material for: For Exercise, Relaxation, or Spirituality: Exploring Participation Motives and Conformity to Masculine Norms among Male and Female Yoga Participants
Source: Int J Environ Res Public Health. 2022 Jan 11;19(2):770. doi: 10.3390/ijerph19020770 (PMC8776006; doi:10.3390/ijerph19020770)
Supplement: Supplementary file 1 [file ijerph-19-00770-s001.zip › ijerph-1543760-supplementary.pdf]

Supplementary Files

Table S1. Structure coefficients for gender canonical function.

Motives	Canonical structure coefficients
	Gender Function
Coping and stress management (COP)	-.629*
Positive affect (PAF)	-.587*
Mind-body integration (MBI)	-.485*
Health and fitness (HFI)	-.484*
Supplementary activity (SUP)	.272
Nimbleness (NIM)	-.251
Competition and social recognition (COM)	.241
Ill-health avoidance (IHA)	-.167
Challenge (CHA)	-.116
Spirituality (SPI)	-.075
Affiliation (AFF)	.059
Weight management and appearance (WMA)	-.011

**Largest absolute correlation between each variable and any discriminant function; Canonical structure coefficient of .30 was considered.*

Table S2. Correlations between age and participation motives (Overall: n = 530).

Variable	Age	PAF	HFI	NIM	MBI	COP	IHA	SPI	CHA	WMA	SUP	AFF	COM
Age	-												
Positive Affect (PAF)	-1.00*	-											
Health and Fitness (HFI)	-.062	.563**	-										
Nimbleness (NIM)	.128**	.431**	.679**	-									
Mind-Body Integration (MBI)	-.201**	.657**	.357**	.132**	-								
Coping and Stress Management (COP)	-.204**	.650**	.432**	.269**	.776**	-							
Ill-Health Avoidance (IHA)	-.007	.364**	.572**	.433**	.310**	.435**	-						
Spirituality (SPI)	-.089*	.360**	.132**	-.046	.707**	.514**	.198**	-					
Challenge (CHA)	-.218**	.536**	.563**	.441**	.459**	.481**	.429**	.259**	-				
Weight Management and Appearance (WMA)	-.114**	.331**	.536**	.434**	.164**	.278**	.568**	.067	.463**	-			
Supplementary Activity (SUP)	-.129**	.070	.262**	.256**	-.044	.055	.195**	-.039	.249**	.215**	-		
Affiliation (AFF)	-.071	.291**	.313**	.213**	.211**	.234**	.315**	.133**	.415**	.340**	.146**	-	
Competition and Social Recognition (COM)	-.175**	.223**	.316**	.248**	.166**	.219**	.335**	.131**	.599**	.503**	.307**	.434**	-

Table S3. Correlations between age and participation motives (Males: *n* = 136).

Variable	Age	PAF	HFI	NIM	MBI	COP	IHA	SPI	CHA	WMA	SUP	AFF	COM
Age	-												
Positive Affect (PAF)	-.035	-											
Health and Fitness (HFI)	-.095	.580**	-										
Nimbleness (NIM)	.087	.352**	.670**	-									
Mind-Body Integration (MBI)	-.178*	.741**	.392**	.060	-								
Coping and Stress Management (COP)	-.190*	.743**	.462**	.199*	.818**	-							
Ill-Health Avoidance (IHA)	-.142	.457**	.550**	.493**	.384**	.570**	-						
Spirituality (SPI)	-.202*	.494**	.221**	-.035	.806**	.626**	.299**	-					
Challenge (CHA)	-.119	.672**	.689**	.464**	.563**	.617**	.489**	.437**	-				
Weight Management and Appearance (WMA)	-.181*	.438**	.655**	.571**	.343**	.476**	.691**	.265**	.573**	-			
Supplementary Activity (SUP)	-.214*	.077	.370**	.278**	-.011	.072	.256**	-.067	.286**	.252**	-		
Affiliation (AFF)	-.101	.482**	.511**	.347**	.409**	.435**	.389**	.338**	.579**	.458**	.128	-	
Competition and Social Recognition (COM)	-.123	.394**	.534**	.405**	.358**	.416**	.541**	.314**	.683**	.614**	.371**	.590**	-

Table S4. Correlations between age and participation motives (Females: *n* = 394).

Variable	Age	PAF	HFI	NIM	MBI	COP	IHA	SPI	CHA	WMA	SUP	AFF	COM
Age	-												
Positive Affect (PAF)	-.108*	-											
Health and Fitness (HFI)	-.024	.526**	-										
Nimbleness (NIM)	.160**	.470**	.679**	-									
Mind-Body Integration (MBI)	-.193**	.575**	.302**	.145**	-								
Coping and Stress Management (COP)	-.189**	.550**	.375**	.285**	.735**	-							
Ill-Health Avoidance (IHA)	.051	.315**	.582**	.404**	.269**	.371**	-						
Spirituality (SPI)	-.041	.292**	.085	-.055	.672**	.473**	.156**	-					
Challenge (CHA)	-.255**	.467**	.505**	.428**	.405**	.415**	.402**	.178**	-				
Weight Management and Appearance (WMA)	-.089	.295**	.500**	.382**	.084	.196**	.524**	-.011	.420**	-			
Supplementary Activity (SUP)	-.116*	.111**	.257**	.266**	-.031	.090	.186**	-.025	.245**	.205**	-		
Affiliation (AFF)	-.062	.205**	.236**	.159**	.123*	.150**	.289**	.047	.344**	.294**	.151**	-	
Competition and Social Recognition (COM)	-.217**	.155**	.236**	.188**	.085	.148**	.253**	.045	.563**	.461**	.274**	.358**	-

Table S5. Correlations between years of practice and participation motives (Overall).

Variable	Years	PAF	HFI	NIM	MBI	COP	IHA	SPI	CHA	WMA	SUP	AFF	COM
Years of Practice	-												
Positive Affect (PAF)	.008	-											
Health and Fitness (HFI)	-.141**	.563**	-										
Nimbleness (NIM)	-.023	.431**	.679**	-									
Mind-Body Integration (MBI)	.057	.657**	.357**	.132**	-								
Coping and Stress Management (COP)	.024	.650**	.432**	.269**	.776**	-							
Ill-Health Avoidance (IHA)	-.030	.364**	.572**	.433**	.310**	.435**	-						
Spirituality (SPI)	.107*	.360**	.132**	-.046	.707**	.514**	,198**	-					
Challenge (CHA)	-.240**	.536**	.563**	.441**	.459**	.481**	.429**	.259**	-				
Weight Management and Appearance (WMA)	-.157**	.331**	.536**	.434**	.164**	.278**	.568**	.067	.463**	-			
Supplementary Activity (SUP)	-.125**	.070	.262**	.256**	-.044	.055	.195**	-.039	.249**	.215**	-		
Affiliation (AFF)	-.073	.291**	.313**	.213**	.211**	.234**	.315**	.133**	.415**	.340**	.146**	-	
Competition and Social Recognition (COM)	-.161**	.223**	.316**	.248**	.166**	.219**	.335**	.131**	.599**	.503**	.307**	.434**	-

Table S6. Correlations between years of practice and participation motives (Males: $n = 136$).

Variable	Years	PAF	HFI	NIM	MBI	COP	IHA	SPI	CHA	WMA	SUP	AFF	COM
Years of Practice	-												
Positive Affect (PAF)	.088	-											
Health and Fitness (HFI)	-.220**	.580**	-										
Nimbleness (NIM)	-.136	.352**	.670**	-									
Mind-Body Integration (MBI)	.135	.741**	.392**	.060	-								
Coping and Stress Management (COP)	.023	.743**	.462**	.199**	.818**	-							
Ill-Health Avoidance (IHA)	-.004	.457**	.550**	.493**	.384**	.570**	-						
Spirituality (SPI)	.128	.494**	.221**	-.035	.806**	.626**	.299**	-					
Challenge (CHA)	-.198*	.672**	.689**	.464**	.563**	.617**	.489**	.437**	-				
Weight Management and Appearance (WMA)	-.158	.438**	.655**	.571**	.343**	.476**	.691**	.265**	.573**	-			
Supplementary Activity (SUP)	-.280**	.077	.370**	.278**	-.011	.072	.256**	-.067	.286**	.252**	-		
Affiliation (AFF)	.074	.482**	.511**	.347**	.409**	.435**	.389**	.338**	.579**	.458**	.128	-	
Competition and Social Recognition (COM)	-.096	.394**	.534**	.405**	.358**	.416**	.541**	.314**	.693**	.614**	.371**	.590**	-

Table S7. Correlations between years of practice and participation motives (Females: n = 394).

Variable	Years	PAF	HFI	NIM	MBI	COP	IHA	SPI	CHA	WMA	SUP	AFF	COM
Years	-												
Positive Affect (PAF)	-.046	-											
Health and Fitness (HFI)	-.139**	.526**	-										
Nimbleness (NIM)	.001	.470**	.679**	-									
Mind-Body Integration (MBI)	.016	.575**	.302**	.145**	-								
Coping and Stress Management (COP)	.003	.550**	.375**	.285**	.735**	-							
Ill-Health Avoidance (IHA)	-.044	.315**	.582**	.404**	.269**	.371**	-						
Spirituality (SPI)	.100*	.292**	.085	-.055	.672**	.473**	.156**	-					
Challenge (CHA)	-.263**	.467**	.505**	.428**	.405**	.415**	.402**	.178**	-				
Weight Management and Appearance (WMA)	-.158**	.295**	.500**	.382**	.084	.196**	.524**	-.011	.420**	-			
Supplementary Activity (SUP)	-.079	.111**	.257**	.266**	-.031	.090	.186**	-.025	.245**	.205**	-		
Affiliation (AFF)	-.117*	.205**	.236**	.159**	.123*	.150**	.289**	.047	.344**	.294**	.151**	-	
Competition and Social Recognition (COM)	-.182*	.155**	.236**	.188**	.085	.148**	.253**	.045	.563**	.461**	.274**	.358**	-

Table S8. Descriptive statistics of Conformity to Masculine Norms subscales by gender and participant subgroups.

	Overall (n = 530)		Male (n = 136)		Female (n = 394)	
	**Teacher**	**Non-Teacher**	**Teacher**	**Non-Teacher**	**Teacher**	**Non-Teacher**
n	**146**	**384**	**45**	**91**	**101**	**293**
**Motives**	**M (sd)**	**M (sd)**	**M (sd)**	**M (sd)**	**M (sd)**	**M (sd)**
Positive Affect (PAF)	4.38** (.69)	4.14 (.98)	4.20* (.77)	3.69 (1.30)	4.46 (.63)	4.29 (.81)
Health and Fitness (HFI)	4.12 (.90)	4.18 .91)	3.87 (1.14)	3.88 (1.18)	4.23 (.76)	4.28 (.87)
Nimbleness (NIM)	4.00 (.99)	4.23* .99)	3.79 (1.22)	4.10 (1.05)	4.10 (1.04)	4.27 (.97)
Mind-Body Integration (MBI)	4.42*** (1.30)	3.72 1.30)	4.27*** (.79)	4.16 (1.56)	4.49*** (.66)	3.89 (1.15)
Coping and Stress Management (COP)	4.11*** (.94)	3.66 1.28)	3.92*** (1.05)	2.97 (1.53)	4.20** (.88)	3.87 (1.11)
Ill-Health Avoidance (IHA)	3.23* (1.25)	2.94 1.35)	3.26* (1.34)	2.68 (1.36)	3.22 (1.22)	3.02 (1.34)
Spirituality (SPI)	3.58*** (1.43)	2.50 1.72)	3.54*** (1.51)	2.30 (1.86)	3.59*** (1.41)	2.56 (1.67)
Challenge (CHA)	2.76 (1.32)	2.80 1.34)	2.77 (1.55)	2.64 (1.46)	2.76 (1.22)	2.84 (1.29)
Weight Management and Appearance (WMA)	2.29 (1.53)	2.46 1.45)	2.36 (1.57)	2.42 (1.48)	2.26 (1.52)	2.47 (1.44)
Supplementary Activity (SUP)	2.22 (1.85)	2.49 1.74)	2.38 (1.80)	2.91 (1.67)	2.15 (1.88)	2.36 (1.74)
Affiliation (AFF)	1.97 (1.46)	1.70 1.43)	2.27* (1.65)	1.62 (1.48)	1.84 (1.36)	1.73 (1.41)
Competition and Social Recognition (COM)	.90 (1.08)	1.02 1.05)	1.10 (1.35)	1.19 (1.24)	.81 (.93)	.97 (.98)

Table S9. Differences in participation motives across participant subgroups (Overall)

Motives	Exercisers	Yogis	Postural Yogis
Positive Affect (PAF)	3.77 (1.34)[a, c]	4.18 (.84)[a, b]	4.57 (.61)[b, c]
Health and Fitness (HFI)	3.96 (.90)[c]	3.90 (1.02)[b]	4.55 (.61)[b, c]
Nimbleness (NIM)	4.28 (.86)[a]	3.73 (1.23)[a, b]	4.53 (.69)[b]
Mind-Body Integration (MBI)	2.54 (1.33)[a, c]	4.28 (.78)[a]	4.40 (.73)[c]
Coping and Stress Management (COP)	2.78 (1.35)[a, c]	2.78 (1.35)[a, b]	3.96 (1.00)[b, c]
Ill-Health Avoidance (IHA)	2.58 (1.27)[c]	2.86 (1.31)[b]	3.44 (1.28)[b, c]
Spirituality (SPI)	.78 (.91)[a, c]	3.68 (1.23)[a, b]	3.20 (1.47)[b, c]
Challenge (CHA)	2.78 (1.24)[c]	2.56 (1.32)[b]	3.33 (1.20)[b, c]
Weight Management and Appearance (WMA)	2.24 (1.46)[c]	2.08 (1.39)[b]	2.84 (1.45)[b, c]
Supplementary Activity (SUP)	2.64 (1.67)[a]	2.16 (1.70)[a]	2.53 (1.88)
Affiliation (AFF)	1.46 (1.39)[c]	1.69 (1.41)[b]	2.06 (1.45)[b, c]
Competition and Social Recognition (COM)	.84 (.95)[c]	.90 (1.02)[b]	1.16 (1.14)[b, c]

[a]*Exercisers ≠ Yogis;* [b]*Yogis ≠ Postural Yogis;* [c]*Exercisers ≠ Postural Yogis*

Table S10. Differences in participation motives across participant subgroups (Males)

Motives	Exercisers	Yogis	Postural Yogis
Positive Affect (PAF)	3.03 (1.38)[a, c]	3.96 (1.01)[a]	4.43 (.70)[c]
Health and Fitness (HFI)	3.75 (.81)[c]	3.40 (1.18)[b]	4.50 (.62)[b, c]
Nimbleness (NIM)	4.26 (.85)[a]	3.35 (1.29)[a, b]	4.49 (.67)[b]
Mind-Body Integration (MBI)	1.89 (1.37)[a, c]	4.16 (.86)[a]	4.17 (.87)[c]
Coping and Stress Management (COP)	2.05 (1.52)[a, c]	3.60 (1.09)[a]	3.95 (1.11)[c]
Ill-Health Avoidance (IHA)	2.44 (1.31)[c]	2.68 (1.29)[b]	3.43 (1.36)[b, c]
Spirituality (SPI)	.64 (1.03)[a, c]	3.65 (1.22)[a]	3.37 (1.59)[c]
Challenge (CHA)	1.92 (1.36)[c]	2.40 (1.45)[b]	3.61 (1.11)[b, c]
Weight Management and Appearance (WMA)	2.06 (1.51)[c]	1.90 (1.29)[b]	3.22 (1.39)[b, c]
Supplementary Activity (SUP)	3.04 (1.51)[a]	2010 (1.60)[a, b]	3.18 (1.84)[b]
Affiliation (AFF)	1.13 (1.35)[c]	1.75 (1.48)[b]	2/49 (1.57)[b, c]
Competition and Social Recognition (COM)	.86 (1.06)[c]	.89 (1.06)[b]	1.68 (1.48)[b, c]

[a]*Exercisers ≠ Yogis;* [b]*Yogis ≠ Postural Yogis;* [c]*Exercisers ≠ Postural Yogis*

Table S11. Differences in participation motives across participant subgroups (Females)

Motives	Exercisers	Yogis	Postural Yogis
Positive Affect (PAF)	3.96 (.89)[a, c]	4.26 (.76)[a, b]	4.61 (.58)[b, c]
Health and Fitness (HFI)	4.06 (.93)[c]	4.07 (.91)[b]	4.57 (.61)[b, c]
Nimbleness (NIM)	4.28 (.86)[a]	3.86 (1.19)[a, b]	4.54 (.70)[b]
Mind-Body Integration (MBI)	2.82 (1.23)[a, c]	4.33 (.74)[a]	4.47 (.68)[c]
Coping and Stress Management (COP)	3.09 (1.14)[a, c]	4.08 (.94)[a]	4.31 (.86)[c]
Ill-Health Avoidance (IHA)	2.64 (1.25)[c]	2.93 (1.31)[b]	3.44 (1.26)[b, c]
Spirituality (SPI)	.84 (.85)[a, c]	3.69 (1.24)[a, b]	3.15 (1.43)[b, c]
Challenge (CHA)	2.43 (1.16)[c]	2.62 (1.28)[b]	3.24 (1.22)[b, c]
Weight Management and Appearance (WMA)	2.32 (1.45)	2.14 (1.43)[b]	2.73 (1.45)[b]
Supplementary Activity (SUP)	2.47 (1.71)	2.18 (1.74)	2.33 (1.85)
Affiliation (AFF)	1.60 (1.39)	1.67 (1.39)	1.93 (1.39)
Competition and Social Recognition (COM)	.83 (.91)	.90 (1.01)	1.01 (.98)

[a]*Exercisers ≠ Yogis;* [b]*Yogis ≠ Postural Yogis;* [c]*Exercisers ≠ Postural Yogis*
